# The analysis of tetracyclines, quinolones, macrolides, lincosamides, pleuromutilins, and sulfonamides in chicken feathers using UHPLC-MS/MS in order to monitor antibiotic use in the poultry sector

**DOI:** 10.1007/s00216-017-0445-0

**Published:** 2017-07-04

**Authors:** Larissa J. M. Jansen, Yvette J. C. Bolck, Janneau Rademaker, Tina Zuidema, Bjorn J. A. Berendsen

**Affiliations:** RIKILT, Wageningen University and Research, Akkermaalsbos 2, 6708WB, P.O. Box 230, 6700 AE Wageningen, The Netherlands

**Keywords:** Antibiotics, Validation, Feathers, Monitoring, LC-MS/MS

## Abstract

In The Netherlands, all antibiotic treatments should be registered at the farm and in a central database. To enforce correct antibiotic use and registration, and to enforce prudent use of antibiotics, there is a need for methods that are able to detect antibiotic treatments. Ideally, such a method is able to detect antibiotic applications during the entire lifespan of an animal, including treatments administered during the first days of the animals’ lives. Monitoring tissue, as is common practice, only provides a limited window of opportunity, as residue levels in tissue soon drop below measurable quantities. The analysis of feathers proves to be a promising tool in this respect. Furthermore, a qualitative confirmatory method was developed for the analyses of six major groups of antibiotics in ground chicken feathers, aiming for a detection limit as low as reasonably possible. The method was validated according to Commission Decision 2002/657/EC. All compounds comply with the criteria and, as a matter of fact, 58% of the compounds could also be quantified according to regulations. Additionally, we demonstrated that a less laborious method, in which whole feathers were analyzed, proved successful in the detection of applied antibiotics. Most compounds could be detected at levels of 2 μg kg^−1^ or below with the exception of sulfachloropyridazine, tylosin, and tylvalosin. This demonstrates the effectiveness of feather analysis to detect antibiotic use to allow effective enforcement of antibiotic use and prevent the illegal, off-label, and nonregistered use of antibiotics.

## Introduction

The use of antibiotics is common practice to treat bacterial infections in the poultry sector. Antibiotic treatments, mostly orally administered through drinking water, should be carried out according to registration in order to prevent excessive antibiotic residues in food products meant for human consumption. Through Commission Regulation 96/23/EC [1], the European Union (EU) strictly monitors the presence of antibiotics in food of animal origin on the basis of EU/37/2010 [2], which establishes maximum residue limits (MRL) for matrices such as muscle, liver, and eggs.

Besides the issue of antibiotic residues in food products through which consumer exposure can occur, there has been a growing concern of antibiotic resistance, which is currently one of the major public health threats [3]. The use of antimicrobial agents is considered to be the most important factor in the selection of resistant bacteria, where superfluous use of antibiotics is often considered to be the main factor [4]. However, also the use of smaller amounts of antibiotics, e.g., as a preventive measure or contamination, can contribute to selection and persistence of resistant bacteria [5, 6].

Recently, The Netherlands has focused on prudent use of antibiotics to fight the increasing incidence of antibiotic resistance [5]. In order to prevent extensive or unnecessary use of antibiotics in The Netherlands, policies have been implemented to restrict the use of antibiotics. These policies require that every antibiotic treatment is recorded at the farm and in a central database. Previously, the antibiotic use could only be enforced through monitoring food products. However, the methods applied in routine monitoring of food products are usually designed to enforce the MRL. Therefore, and because of the high excretion rates in life animals, these procedures have a short detection window: they usually will only be able to detect antibiotics used until the final days before slaughter. Especially when antibiotics are administered early in the animal’s life, no residues are expected to be detected in food products. This indicates that regular analysis of food products at the MRL, while useful for monitoring for safety and certain good agricultural practice, is not suitable for the detection and prevention of nonregistered use of antibiotics.

There is a need for methods that are able to detect the use of antibiotics administered over the entire life span of an animal, including treatments administered during the first days of the animals’ lives. In previous research focusing on the excretion of oxytetracycline [7], multiple fluoroquinolones (enrofloxacin, its metabolite ciprofloxacin [8], and flumequin [9]) and florfenicol and its metabolite florfenicol amine [10] to feathers, it has already been shown that antibiotic residues can still be detected in feathers long after treatment. In another study, different antibiotics in feather meal, originating from different countries, were detected, including antibiotics that are registered as banned substances in the country of origin [11]. These findings support the claim that feathers are a promising matrix for monitoring antibiotic use in the poultry sector.

In the research investigating the excretion of oxytetracyline to feathers, it was suggested that after oral treatment, antibiotics enter the bloodstream and disposite into the rachis of feathers [7]. It was suggested that concurrently, the antibiotics can exit the body by excretion through the uropygial gland and are dispositioned on the feathers through grooming behavior [8].

Recently, more in-depth research on the disposition of antibiotics to feathers was done in order to explore the future possibilities of the use of this matrix for monitoring purposes [12]. This study involved segmentation of incurred feathers containing enrofloxacin, confirming that antibiotics are incorporated inside the feathers after oral treatment, as was already suggested by earlier results found based on segmentation of incurred feathers containing oxytetracycline [7]. This mechanism allows discrimination of different exposure routes and provides promising results for antedating antibiotic treatments.

In order to effectively monitor antibiotic use in the poultry sector, a broad confirmatory method is needed, covering multiple groups of antibiotics, applicable to feathers. To our knowledge, this is the first time a multi-residue method for the qualitative confirmatory analysis of tetracyclines, quinolones, macrolides, lincosamides, pleuromutilins, and sulfonamides in chicken feathers, using ultra-high performance liquid chromatography (UHPLC) coupled to tandem mass spectrometry (MS/MS) is presented. The method was fully validated according to Commission Decision 2002/657/EC [13]. The method and validation characteristics are presented here. Note that to cover an even wider range of relevant compounds, multiple analytical methods can be applied.

## Materials and methods

The following antibiotics are referred to when different antibiotic classes are mentioned. Tetracyclines: chlortetracycline, oxytetracycline, tetracycline, and doxycycline. (Fluoro)quinolones: ciprofloxacin, danofloxacin, difloxacin, enrofloxacin, flumequin, marbofloxacin, nalidixic acid, norfloxacin, oxolic acid, and sarafloxacin. Macrolides: erythromycin, gamithromycin, josamycin, natamycin, neospiramycin, spiramycin, tildipirosin, tilmicosin, tulathromycin, tylosin, and tylvalosin. Lincosamides: lincomycin and pirlimycin. Pleuromutilins: tiamulin and valnemulin. Sulfonamides: dapsone, sulfacetamide, sulfachloropyridazine, sulfadiazine, sulfadimethoxine, sulfadimidine, sulfadoxine, sulfamerazine, sulfamethizole, sulfamethoxazole, sulfamethoxypyridazine, sulfamoxole, sulfaphenazole, sulfapyridine, sulfauqinoxaline, sulfathiazole, sulfisoxazole, and sulfamonomethoxine.

### Reference standards

The reference standards of chlortetracycline, oxytetracycline, tetracycline, ciprofloxacin, danofloxacin, difloxacin, enrofloxacin, flumequin, marbofloxacin, nalidixic acid, norfloxacin, oxolinic acid, sarafloxacin, erythromycin, josamycin, lincomycin, spiramycin, tiamulin, tylosin, valnemulin, dapson, sulfacetamide, sulfachlorpyridazine, sulfadimethoxine, sulfadimidine, sulfadoxine, sulfamerazine, sulfamethizole, sulfamethoxazole, sulfamethoxypyridazine, sulfamoxole, sulfaphenazole, sulfapyridine, sulfaquinoxaline, sulfathiazole, and sulfisoxazole were purchased at Sigma-Aldrich (St. Louis, MO, USA). Neospiramycin, pirlimycin, and natamycin were purchased at Toronto Research Chemicals (Toronto, ON, Canada). Doxycycline and sulfadiazine were purchased at Council of Europe (EDQM, Strasbourg, France). Gamithromycin and tulathromycin were purchased at Santa Cruz Biotechnology (Dallas, TX, USA). Tilmicosin was purchased at Dr. Ehrenstorfer GMBH (Augsburg, Germany), tylvalosin at ECO Animal Health (London, UK), tildipirosin at MSD Animal Health (Boxmeer, The Netherlands), and sulfamonomethoxine at TCI Europe (Zwijndrecht, Belgium).

The internal standards norfloxacin-d_5_, ciprofloxacin-d_8_, enrofloxacin-d_5_, sarafloxacin-d_8_, difloxacin-d_3_, oxolinic acid-d_5_, nalidic acid-d_5_, flumequin-^13^C_3_, sulfathiazole-^13^C_6_, sulfapyridine-^13^C_6_, sulfamerazine-^13^C_6_, sulfadimidine-^13^C_6_, sulfamethizole-^13^C_6_, sulfachlorpyridazine-^13^C_6_, sulfadoxine-d_3_, sulfisoxazole-^13^C_6_, sulfadimethoxine-d_6_, and sulfaquinoxaline-^13^C_6_ were purchased at Witega (Berlin, Germany). Erythromycin-^13^C-d_3_, spiramycin-d_3_, lincomycin-d_3_, sulfadiazine-d_4_, and dapsone-d_8_ were purchased at Toronto Research Chemicals. Tetracycline-d_6_ and gamithromycin-d_4_ were purchased at Santa Cruz Biotechnology. Demeclocycline was purchased at Sigma-Aldrich and tildipirosin-d_10_ at MSD Animal Health.

### Reagents

Methanol ULC/MS grade (MeOH) and acetonitrile ULC/MS grade (ACN) were purchased at Actu-All Chemicals (Oss, The Netherlands). Formic acid (FA), citric acid monohydrate, sodium hydroxide (NaOH), and disodium hydrogen phosphate dihydrate were purchased at VWR International (Darmstadt, Germany). Ammonium hydroxide (25%) was purchased at Merck Millipore (Darmstadt, Germany). Trifluoroacetic acid (TFA) was purchased at Sigma-Aldrich. Milli-Q water, referred to as water from here on, was prepared using a Milli-Q system with a resistivity of at least 18.2 M Ω cm^−1^ (Merck Millipore). McIlvain-ethylenediaminetetraacetic acid (EDTA) buffer was prepared by dissolving 74.4 g disodium EDTA (VWR International) in 500 mL 0.1 M citric acid and 280 mL 0.2 M phosphate buffer. The pH was adjusted to 4.0 by adding 0.1 M citric acid or 0.2 M phosphate buffer. The total volume was adjusted to 2 L.

Stock solutions of the reference standards and internal standards were made at 1000 mg L^−1^ for tetracyclines, macrolides, lincosamides, pleuromutilins, and sulfonamides and at 100 mg L^−1^ for quinolones. Tetracyclines and sulfonamides were dissolved in MeOH, quinolones in 2% 2M ammonia hydroxide in MeOH, lincosamides, tylosin, tiamulin, and valnemulin in water, tildipyrosin and natamycin in MeOH, and the remainder of the macrolides and pleuromutilins in ACN.

A mixed solution of reference standards was made at 0.5/0.1 mg L^−1^ (tetracyclines, quinolones, macrolides, lincosamides, pleuromutilins/sulfonamides) in MeOH and a mixed solution of internal standards was made at 0.5 mg L^−1^ for all compounds in MeOH.

### Analysis procedure

**Fig. 1:**
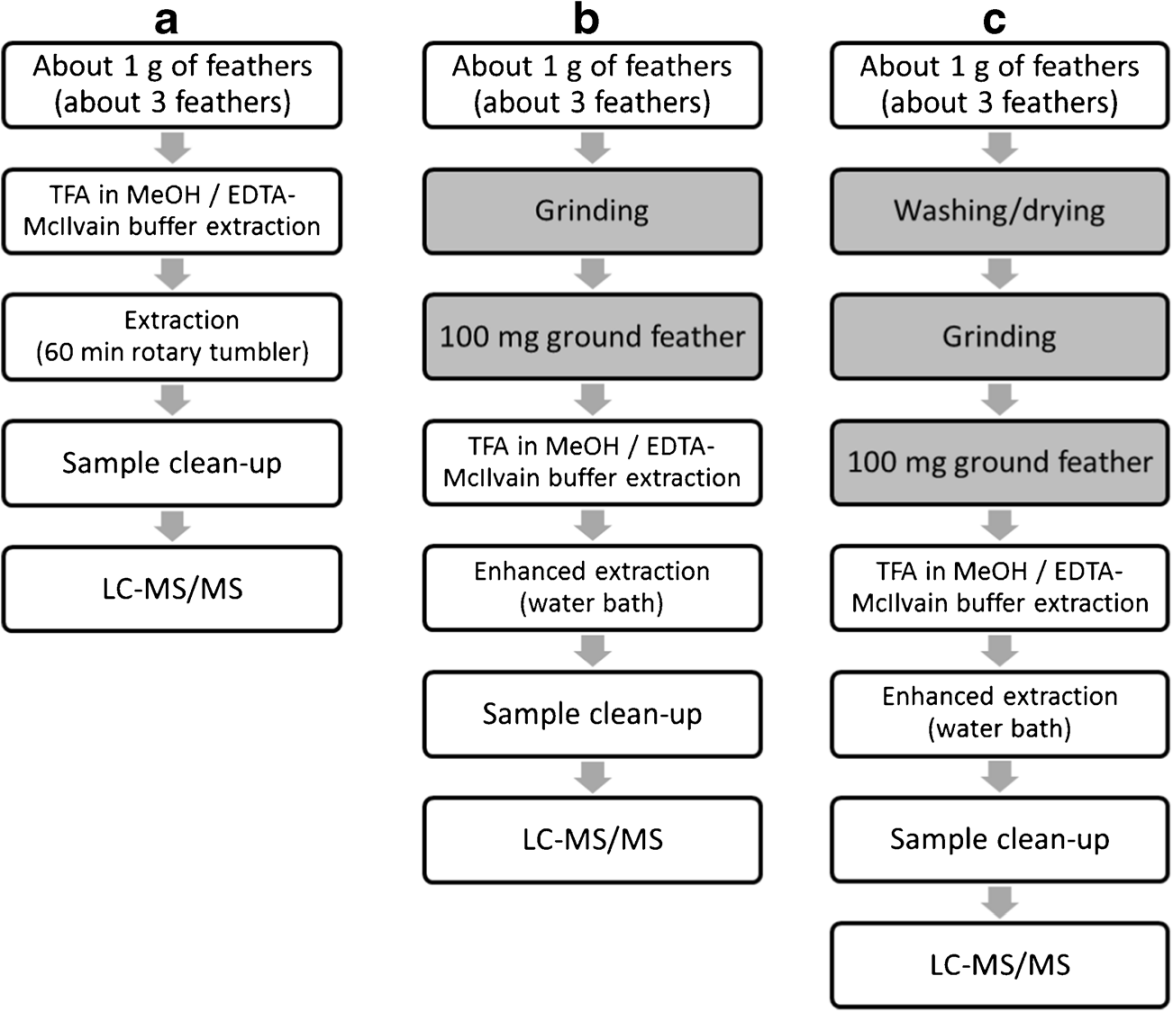
Overview of the method for freely extractable residues (**a**), for total residues (**b**), and for non-freely extractable residues (**c**), where grey boxes indicate the additional steps needed compared with approach (**a**)

The main purpose of analyzing feathers is to determine whether and which antibiotic residues are present. A distinction can be made between freely extractable and non-freely extractable residues if required. Freely extractable residues are those that can be extracted from whole feathers, as opposed to not freely extractable residues that can only be extracted after grinding, which indicates they are incorporated inside the feather [12]. The validated method is designed to allow three options. The first option is to extract whole feathers yielding only the freely extractable residues. This approach is simple and fast and is useful to efficiently and effectively detect what antibiotics are present. The second option is to grind the whole feathers before extraction, yielding the total amount of antibiotic residues. This approach is especially relevant if the total antibiotic concentration is to be determined. Last, the non-freely extractable residues can be determined by grinding the feathers after a washing procedure of the whole feathers has been applied. This procedure is most laborious but is mandatory if treatments should be antedated using a segmentation procedure [7, 12]. A schematic overview of the individual steps for the different applications is displayed in Fig. 1.

### Sample extraction

#### Detection of freely extractable residues

Weigh 1 g of feathers (approximately three large wing feathers) into a 50 mL polypropylene (PP) centrifuge tube (Greiner Bio-One, Alphen aan de Rijn, The Netherlands). Add internal standard solution and 2 mL of 0.125% TFA in MeOH to all samples. Shake thoroughly by hand and add 16 mL of McIlvain-EDTA buffer. Shake for 60 min using a rotary tumbler (Heidolph REAX-2, Schwabach, Germany) and centrifuge for 5 min at 3500 g. From here, extracts are submitted to sample clean-up.

#### Detection of total residues

Transfer 1 g of feather sample, cut to fit using secateurs, into a zirconium grinding bucket. Add a zirconium grinding ball (20 mm diameter) and grind two times for 4 min using a mixer mill (Type MM301, Retsch Haan, Germany). Weigh 100 mg of ground feather into a 12 mL PP centrifuge tube. Add internal standard solution to each sample and wait for 5 min. Add 2 mL 0.125% TFA in MeOH and place the centrifuge tubes in a water bath at 45 °C during 60 min. Subsequently, add 2 mL of McIlvain-EDTA buffer, shake for 5 min using a rotary tumbler and centrifuge at 3500 g for 10 min. Decant the supernatant into a 50 mL PP centrifuge tube and dilute with 14 mL McIlvain-EDTA buffer. From here, extracts are submitted to sample clean-up.

#### Detection of non-freely extractable residues

The analysis of non-freely extractable residues is similar to the procedure for analysis of total residues, but is preceded by a washing procedure. Weigh 1 g feathers (approximately three large whole feathers) into a 50 mL PP) centrifuge tubes. Add 20 mL 0.125% TFA in MeOH and wash the feathers by shaking for 5 min using a rotary tumbler. Decant the washing solvent and repeat this procedure two more times and combine the wash solvent fractions. Dry the washed feathers overnight at room temperature and continue the procedure for analysis of total residues.

### Sample clean-up

Condition a Strata-X reversed-phase polymeric SPE cartridge (Phenomenex, Torrance, CA, USA) with 5 mL MeOH and subsequently 5 mL water. Transfer the complete extract onto the cartridge and slowly pass it through (if needed by applying vacuum) to allow interaction between the SPE material and the antibiotic residues. Rinse the cartridges with 5 mL of water and dry by applying vacuum for 1 min. Elute the residues with 5 mL MeOH into a 14 mL glass tube. Evaporate the solvent (40 °C, N_2_) using a TurboVap LV Evaporator (Zymark, Hopkinton, MA, USA) and reconstitute the residues in 200 μL MeOH by using a vortex mixer (IKA, Staufen, Germany). Dilute with 300 μL water and transfer the extract into a glass vial suitable for LC-MS/MS analysis.

### UHPLC-MS/MS

The UHPLC system consists of an Acquity model (Waters, Milford, MA, USA) with an Acquity HSS-T3 C_18_ analytical column of 2.1 × 100 mm, 1.7 μm (Waters), placed in a column oven at 30 °C. The mobile phase consists of 2 mM ammonium formate and 0.16% FA in water (Solvent A) and 2 mM ammonium formate and 0.16% FA in MeOH (Solvent B). The gradient: 0–1.0 min, 0% mobile phase B, 1.0–2.5 min, linear increase to 25% B, 2.5–5.4 linear increase to 70% B, and 5.4–5.5 min linear increase to 100% with a final hold of 1.0 min. The gradient is returned to its initial conditions within 0.1 min and the column is allowed to equilibrate for 0.9 min before the next injection is initiated, resulting in a total run of 7.5 min. The flow rate is 0.4 mL min^−1^ and the injection volume is 5 μL. Detection is carried out by MS/MS using a Xevo TQS (Waters) in positive electrospray ionization (ESI) mode. The operating parameters are: capillary voltage, 3.0 kV; source temperature, 130 °C; desolvation temperature, 450 °C; cone gas flow, 150 L h^−1^; and desolvation gas, 650 L h^−1^. The antibiotics were fragmented using collision induced dissociation (argon). SRM transitions were selected based on the abundance of the signal and, if multiple options were available, the selectivity of the transition [14] (Table 1). Data were acquired and processed using MassLynx 4.1 software (Waters).

**Table 1:** SRM transitions of the validated compounds

Compound	Precursor ion	Product ion^a^	Cone (V)	Collision energy (eV)
Chlortetracycline	478.9	153.9 444.0	2	26 20
Tetracycline	444.9	410.0 153,9	2	18 26
Tetracycline d_6_	450.9	416.0	2	18
Oxytetracycline	460.9	426.0 200,9		16 36
Demeclocycline	465.3	430.2	2	20
Doxycycline	444.9	428.0 320.9	2	20 30
Marbofloxacin	363.0	319.9 72.0	20	14 33
Norfloxacin	320.0	231.0 282.0	20	19 27
Norfloxacin d_5_	325.1	231.0	20	37
Ciprofloxacin	332.0	231.0 288.0	20	33 17
Ciprofloxacin d_8_	340.1	235.0	20	34
Danofloxacin	358.0	82.0 255.1	20	55 37
Danofloxacin d_3_	361.0	85.0	20	55
Enrofloxacin	360.1	316.0 286.0	20	18 31
Enrofloxacin d_5_	365.1	321.0	20	18
Sarafloxacin	386.1	342.0 299.0	20	18 25
Sarafloxacin d_8_	394.0	350.0	20	18
Difloxacin	400.0	356.0 299.0	20	18 26
Difloxacin d_3_	403.0	359.0	20	19
Nalidixic acid	233.1	215.2 187.1	25	15 25
Nalidixic acid d_5_	238.1	220.2	25	15
Oxolinic acid	262.1	160.2 244.2	25	35 20
Oxolinic acid d_5_	267.1	249.2	25	20
Flumequine	262.1	244.2 202.1	25	20 30
Flumequine ^13^C_3_	265.1	247.2	25	20
Tildipirosin	368.0	98.3 174.2	25	20 20
Tildipirosin d_10_	372.9	108.0	25	15
Tulathromycin	404.0	72.1 158.3	25	38 20
Lincomycin	407.1	126.1 359.0	25	22 17
Lincomycin d_3_	410.1	129.1	25	23
Spiramycin I	422.3	101.3 174.1	25	20 18
Spiramycin I d_3_	423.7	174.0	25	18
Neospiramycin I	699.4	174.1 142.1	10	25 20
Pirlimycin	411.0	112.0 363.1	25	21 15
Tilmicosin	435.3	98.9 143.2	25	17 15
Gamithromycin	777.5	116.4 158.0	25	31 31
Gamithromycin d_4_	781.5	158.0	25	31
Tiamulin	494.1	192.1 119.0	25	18 37
Erythromycin	734.2	158.2 576.4	25	25 15
Erythromycin C_13_ d_3_	738.2	162.0	25	25
Tylosin	916.2	174.0 772.2	25	31 29
Valnemulin	565.1	263.1 164.1	25	17 29
Josamycin	828.1	109.0 174.0	25	55 27
Tylvalosin	1042.2	109.3 174.0	25	30 34
Natamycin	666.3	485.3 503.3	25	5 25
Sulfadiazine	251.0	156.0 91.9	25	13 23
Sulfadiazine d_4_	255.0	160.0	25	15
Sulfacetamide	215.1	92.0 155.9	25	18 15
Sulfapyridine	250.2	91.9 155.9	25	30 15
Sulfapyridine ^13^C_6_	256.2	97.9	25	30
Sulfathiazole	256.0	155.9 91.9	25	14 23
Sulfathiazole ^13^C_6_	262.0	161.9	25	14
Sulfamerazine	265.0	91.9 155.9	25	30 15
Sulfamerazine ^13^C_6_	271.0	97.9	25	25
Dapsone	249.0	155.9 107.9	25	13 19
Dapsone d_8_	257.0	160.0	25	14
Sulfamoxole	268.2	156.1 92.2	25	13 25
Sulfamethizole	271.0	155.9 92.0	25	13 25
Sulfamethizole ^13^C_6_	277.0	162.0	25	14
Sulfadimidine	279.0	186.0 124.0	25	15 21
Sulfadimidine ^13^C_6_	285.0	185.9	25	17
Sulfamethoxypyridazine	281.0	156.0 92.0	25	15 30
Sulfamethoxypyridazine d_3_	284.0	155.9	25	15
Sulfamethoxazole	254.0	155.9 92.0	25	14 18
Sulfamethoxazole d_4_	258.0	160.0	25	15
Sulfisoxazole	268.0	156.0 92.0	25	13 30
Sulfisoxazole ^13^C_6_	274.0	162.0	25	30
Sulfamonomethoxine	281.2	156.1 92.2	30	20 30
Sulfachloropyridazine	284.9	155.9 91.9	25	14 26
Sulfachloropyridazine ^13^C_6_	290.9	161.9	25	14
Sulfadoxine	311.1	155.9 92.0	25	17 30
Sulfadoxine d_3_	314.0	155.9	25	14
Sulfaquinoxaline	301.0	155.9 92.0	25	15 27
Sulfaquinoxaline ^13^C_6_	307.0	161.9	25	15
Sulfadimethoxine	311.1	156.1 92.0	25	15 27
Sulfadimethoxine d_6_	317.0	162.1	25	21
Sulfaphenazole	315.0	92.0 155.9	25	19 26

^a^ Underlined values are the quantifier ion.

### Method validation

Although the method was designed as a qualitative confirmatory method, a full validation was performed according to quantitative confirmatory criteria as described in Commission Decision 2002/657/EC, which implements Council Directive 96/23/EC concerning the performance of analytical methods and the interpretation of results. The full validation was carried out using the worst-case scenario: determination of total residue as described above as this will result in the most challenging sample extracts (excluding washing and including grinding). The following parameters related to a qualitative confirmatory method were determined: selectivity, stability, confirmation of the identity, decision limit (CCα), and detection capability (CCβ). Limit of detection (LOD) and limit of confirmation (LOC) were additionally determined for the analysis of freely extractable residues. In order to critically assess the applicability of the method, quantitative confirmatory parameters were assessed additionally. These include trueness, repeatability (RSD_r_), repeatability including matrix variation (RSD_r*_), within-laboratory reproducibility (RSD_RL_), and linearity.

The validation was carried out using blank chicken feather samples from different origins (n = 21). Ultimately, a validation is carried out using incurred certified reference materials. Because these are not available for this specific application, the second best option was used: spiking. Since there is no MRL or other target level for antibiotics in feathers, target levels were chosen based on MRLs in other animal matrices and the performance of the method during method development. The target level for tetracyclines, quinolones, macrolides, lincosamides, and pleuromutilins was set at 100 μg kg^−1^ and for sulfonamides at 20 μg kg^−1^. The validation was carried out at three different levels (0.5, 1.0, and 1.5 * target level), on three different occasions by two different technicians.

Quantitative results were obtained through correction with the corresponding isotopically labeled internal standards, if available. In case no isotopically labeled internal standard was available, corrections were made using another internal standard. Chlortetracycline, oxytetracycline, and doxycycline were corrected utilizing demeclocycline; marbofloxacin using norfloxacin-d_5_; josamycin, natamycin, tiamulin, and valnemulin using erythromycin-^13^C-d_3_; tylosin, neospiramycin I, pirlimycin, tilmicosin, and tylvalosin using gamithromycin-d_4_; tulathromycin using lincomycin-d_3_; sulfamonomethoxine using sulfisoxazole- ^13^C_6_, and sulfamoxole, sulfacetamide, and sulfaphenazole using sulfadimethoxine-d_6_.

In the stage of method development, it was already determined that when analyzing the total residues, chlortetracycline and natamycin could not be detected at relevant concentration levels. For this reason, they were excluded from the method validation.

#### Selectivity

In order to determine the selectivity, 21 blank samples were analyzed without addition of any reference standards except the internal standards. Selectivity was assessed by checking the signal of the blank materials for interferences at the retention times corresponding to the antibiotics.

#### Stability

Stability data for the antibiotics in solution were reported previously [15]. Stability in matrix was determined by storing one of the validation series at −80 °C for 28 d.. After 28 d, the samples were re-analyzed and results were re-evaluated based on the validation criteria.

#### Decision limit (CCα) and detection capability (CCβ)

For the qualitative analysis of each compound, CCβ was set at the spiking concentration at which in at least 95% of the samples the identity of the compound was confirmed. Per definition, CCα is below this value. For compounds that can be analyzed quantitatively, CCα and CCβ were estimated using the linearity approach as stated in CD 2002/657/EC [13] according to the procedures established in ISO 11843 [16]. The resulting outcomes were visually verified based on the chromatograms of the samples spiked at the lowest validation level.

#### Limit of detection (LOD) and limit of confirmation (LOC)

For the analysis of freely extractable residues, the LOD and LOC were determined by analyzing samples spiked at 0.02, 0.05, and 0.1 * target level on four different occasions. For this assessment, three different LC-MS/MS systems that are regularly used for analysis of feathers were applied, including a Waters Xevo TQS systems and two AB Sciex QTrap 6500 (Framingham, MA, USA) systems. The LOD is established as the concentration level at which the response of the most intense ion was still detectable (S/N > 3). The LOC was set at the concentration level at which the least abundant diagnostic ion was still detectable (S/N > 3). For both parameters, a worst case approach was chosen, so the LOD and LOC are well achievable on all occasions.

#### Confirmation of identity

In 2002/657/EC, criteria were established for the allowed deviation of the relative abundance of both diagnostic ions (ion ratio) resulting from an unknown sample. These criteria are summarized in Table 2. Note that these criteria are currently under debate [17]. Furthermore, the relative retention time of an antibiotic should not deviate more than 2.5% from the reference relative retention time. In order to assess the possibility to confirm the identity of a detected compound using the presented method, the average ion ratio and the average relative retention time of the matrix matched calibration samples were used as the reference value. To comply with the criteria established for a confirmatory analysis, over 95% of the validation samples should comply with these confirmatory criteria.

**Table 2:** Criteria for the confirmation of the identity according to CD 2002/657/EC [13]

Ion ratio reference (R)	Allowed deviation ion ratio of unknown sample
R> 50%	≤20%
20% < R ≤ 50%	≤25%
10% < R ≤ 20%	≤30%
R ≤ 10%	≤50%

#### Trueness, repeatability, selectivity, and within-lab reproducibility

During one day, seven different blank batches of ground chicken feathers were spiked at three levels: 50, 100, and 150 μg kg^−1^ for tetracyclines, quinolones, macrolides, lincosamides, and pleuromutilins, and 10, 20, and 30 μg kg^−1^ for sulfonamides. The use of different blank feather batches during validation results in a better understanding of the between-sample variation and will reflect a routine analysis situation better. Therefore, the calculated repeatability will include matrix variation, which will be indicated as RSD_r*_. In order to also determine the true repeatability (RSD_r_) as described in 2002/657/EC [13] (being the repeatability within a single batch), additionally seven samples of the same batch were spiked at target level.

The entire procedure was carried out on three different occasions, which adds up to a total of 21 feather sample batches obtained over three different days at each of the concentration levels. Response factors were calculated by dividing the area of the most abundant product ion of the compound by the area of the internal standard. Trueness, repeatability, and within-lab reproducibility were calculated using analysis of variance (ANOVA).

The performance criteria were established based on the target values, meaning that in this case trueness must lie between 80 and 110%. According to 2002/657/EC, the relative within-lab reproducibility (RSD_RL_) is considered to be acceptable if below the value calculated from the Horwitz equation [18]. However, as demonstrated by Thompson [19], the Horwitz equation is not applicable to the lower concentration range (<120 μg kg^−1^) and therefore a complementary model was suggested. We adopted these more stringent criteria. Following the complementary model, the RSD_RL_ for the levels <120 μg kg^−1^ should be below 22%. Calculated using the Horwitz equation, the RSD_RL_ for the target level 150 μg kg^−1^ should be below 21.1%. The RSD_r_ is found acceptable if below two-thirds of the RSD_RL_, meaning that for levels <120 μg kg^−1^, RSD_r_ should be below 14.7% and below 14.1% at 150 μg kg^−1^. For RSD_r*_ the same criteria were applied as for RSD_r_.

#### Linearity

On three different days a matrix matched calibration line was prepared at 0, 0.5, 1.0, 2.0, and 3.0 * target level by adding solutions of the antibiotics to aliquots of a ground blank chicken feather sample (a different batch than the seven that were used for determination of trueness, RSD_r_, RSD_r*_, and RSD_RL_. Calibration lines were constructed by plotting the response factors versus the added concentration and carrying out least squares linear regression. The linearity was considered acceptable if the coefficient of correlation was at least 0.990.

### Analysis of feathers samples

Feather samples were collected in the slaughter phase. These were analyzed to assess the presented approach. In this case, the method for detection of freely extractable residues was applied as described above. A matrix matched calibration curve was prepared by spiking at 0, 0.5, 2.5, 5, 15, and 25 ng to 1 g of whole feathers for sulfonamides and 0, 2, 10, 20, 60, and 100 ng to 1 g of whole feathers for tetracyclines, quinolones, macrolides, lincosamides, and pleuromutilins. LC-MS/MS analysis for this study was carried out using UHPLC parameters as described above. Detection was done using an AB Sciex Q-Trap 6500 mass spectrometer in the positive electrospray ionization (ESI) mode. The operating parameters were: capillary voltage, 2.0 kV; cone voltage, 25 V; source offset, 20 V; source temperature, 150 °C; desolvation temperature, 550 °C; cone gas flow, 150 L h^−1^; and desolvation gas, 600 L h^−1^. Data were acquired and processed using MultiQuant 2.02 software (AB Sciex).

During this research, all procedures regarding human and animal rights were followed.

## Results and discussion

### Washing versus extraction procedure

In earlier research investigating the distribution of enrofloxacin and ciprofloxacin to feathers [12], it was already shown that in order to discriminate extractable and non-extractable residues, a similar solvent should be used for both washing and extraction, because when washing with a similar solvent as used for extraction, it can be stated that the residues obtained during extraction after washing originate from non-extractable residues, only accessible after grinding. Therefore, based on the results and this assumption, the best washing solvent for this purpose is 0.125% TFA in MeOH.

### LC-MS/MS

The LC method applied for analysis of the antibiotics in this application is a very generic separation applying common mobile phases and gradient elution. Although our research showed that the selection of the UHPLC column was not very critical [20], a universal column that was especially designed to retain both polar and non-polar compounds and which is compatible with 100% aqueous mobile phases was used. This yielded somewhat higher retention of some polar compounds.

The detection was carried out using tandem MS in Multiple Reaction Monitoring mode. The precursor ions and product ions were determined by continuous infusion of the individual compounds and the ionization setting were optimized. The selection of product ions was done based on their abundance (to allow low detection limits) in combination with their selectivity (in case multiple product ions showing sufficiently high signals were observed) [14].

### Validation

The aim of the validation was to assess the qualitative confirmatory aspect of the method, and additionally the quantitative aspect was evaluated. The results for trueness, repeatability, within-laboratory reproducibility, confirmation of the identity, CCα, and CCβ are presented in Table 3. Confirmation of the identity is expressed as the percentage of positively confirmed samples out of the 21 spiked samples (seven per validation level on three different occasions). As a result of an outlying result, most probably caused during spiking, most results are based on 20 results instead of 21. In case of tulathromycin, the results are based on a 1-d validation and additional data will be collected during future analysis.

**Table 3:** Determined trueness, repeatability, within-laboratory reproducibility, CCα, CCβ, and confirmatory performance as determined during the validation; n is the number of samples used for the calculations and is only presented if <21

Analyt	Levels (μg kg^−1^)	Trueness (%)	RSD_r_ (%)	RSD_r*_ (%)	RSD_RL_ (%)	Identity confirmed (%)	CCα^a^ (μg kg^−1^)	CCβ^a^ (μg kg^−1^)
Tetracyclines								
Doxycycline	50_(n=20)_	103		17.1	18.7	100		
	100	89	5.6	16.3	20.9	100	<50	50
	150_(n=20)_	85		20.4	24.6	100		
Oxytetracycline	50	99		17.4	19.7	100		
	100	95	6.3	12.1	13.0	100	<50	50
	150_(n=20)_	87		16.4	16.5	100		
Tetracycline	50_(n=20)_	106		8.0	14.2	100		
	100	98	5.2	11.8	21.3	100	40	50
	150_(n=20)_	94		9.1	21.3	100		
Quinolones								
Ciprofloxacin	50_(n=20)_	93		8.5	8.7	100		
	100	96	6.2	3.9	5.2	100	20	50
	150_(n=20)_	98		7.4	9.0	100		
Danofloxacin	50_(n=20)_	95		14.1	15	100		
	100	97	4.7	7.3	7.6	100	30	50
	150_(n=20)_	96		9.1	9.4	100		
Difloxacin	50_(n=20)_	99		13.5	14	95		
	100	104	4.8	12	12.5	95	50	50
	150_(n=20)_	99		10.7	12.5	95		
Enrofloxacin	50_(n=20)_	98		8.8	10	100		
	100	101	3.8	6.1	6.3	100	30	50
	150_(n=20)_	102		7.3	9.6	100		
Flumequin	50_(n=20)_	103		8.5	11.6	100		
	100	103	12.2	3.5	4.3	100	30	50
	150_(n=20)_	99		7.1	9.6	100		
Marbofloxacin	50	114		29.2	38.3	100		
	100	122	6.8	28.8	37.9	100	<50	50
	150	131		29.8	38.2	100		
Nalidixic acid	50_(n=20)_	102		8.7	12.1	90		
	100	102	1.7	6.7	7.4	86	30	60
	150_(n=20)_	99		7.5	8.7	86		
Norfloxacin	50_(n=20)_	95		15.9	17.5	62		
	100	96	4.2	10.9	11.5	76	40	80
	150_(n=20)_	98		10.3	10.8	95		
Oxolic acid	50_(n=20)_	106		18.8	19.5	100		
	100	110	11.2	12.3	12.7	100	<50	50
	150_(n=20)_	106		11.3	13.3	100		
Sarafloxacin	50_(n=20)_	99		11.8	14	100		
	100	104	6.9	5.7	10.2	100	30	50
	150_(n=20)_	103		7.1	11.6	100		
Macrolides								
Erythromycin	50_(n=20)_	109		11.5	11.8	100		
	100	106	8.9	7.1	8	100	30	50
	150_(n=20)_	103		6.1	8.8	100		
Gamithromycin	50_(n=20)_	116		27	29.2	100		
	100	108	3.3	8.3	12.4	100	<50	50
	150_(n=20)_	106		14.6	14.6	100		
Josamycin	50_(n=20)_	134		51.3	53.8	100		
	100	120	4.9	38.1	38.7	100	<50	50
	150_(n=20)_	125		38.3	38.5	100		
Neospiramycin	50_(n=20)_	113		24.8	26	100		
	100	116	6.6	31	31.8	100	<50	50
	150_(n=20)_	109		29.7	35	95		
Spiramycin	50	104		17.4	22.4	100		
	100	107	1.8	4.5	7.5	100	40	50
	150_(n=20)_	105		9.1	9.1	100		
Tildipirosin	50_(n=20)_	108		15.3	25.2	100		
	100	98	5.7	10.7	18	100	<50	50
	150_(n=20)_	99		20.4	23.5	100		
Tilmicosin	50_(n=20)_	77		39.8	50.8	95		
	100	73	15.2	37.5	41.1	100	<50	50
	150_(n=20)_	88		81.7	87.9	100		
Tulathromycin	50_(n=7)_	85		45.3	72.5	100		
	100_(n=7)_	82	7.3	31.3	50.1	100	<50	50
	150_(n=7)_	112		66.3	106.1	100		
Tylosin	50_(n=20)_	87		26.6	29.9	100		
	100	86	25.8	26.2	26.5	100	<50	50
	150_(n=20)_	94		60.1	63.5	100		
Tylvalosin	50_(n=20)_	118		50.9	53.4	100		
	100	101	27	23.3	23.7	100	<50	50
	150_(n=20)_	120		75.8	78.9	100		
Lincosamides								
Lincomycin	50_(n=20)_	105		7.5	7.9	100		
	100	106	3.7	3.6	3.6	100	20	50
	150_(n=20)_	105		5.3	9.4	100		
Pirlimycin	50_(n=20)_	110		22.4	30.6	100		
	100	107	26.5	20	31.2	100	<50	50
	150_(n=20)_	99		21.6	33.9	100		
Pleuromutilins								
Tiamulin	50_(n=20)_	129		35.9	36.1	100		
	100	119	9.8	22.5	22.6	100	<50	50
	150_(n=20)_	125		26.6	27.5	100		
Valnemulin	50_(n=20)_	123		34.5	39.2	100		
	100	103	10.6	19.8	25.5	100	<50	50
	150_(n=20)_	109		32.6	34.8	100		
Sulfonamides								
Dapsone	10	102		16.2	17.2	100		
	20	102	2.3	4.4	7.0	100	5	10
	30_(n=20)_	99		3.8	10.2	100		
Sulfacetamide	10	101		17	18.1	95		
	20	104	3.7	9.0	9.0	100	<10	10
	30_(n=20)_	97		20	22.6	100		
Sulfachloropyridazine	10	103		16.0	16.6	100		
	20	105	6.5	5.1	5.2	100	5	10
	30_(n=20)_	104		6.0	9.4	100		
Sulfadiazine	10	104		15.6	15.8	100		
	20	103	1.4	4.0	4.6	100	6	10
	30_(n=20)_	102		5.7	8.7	95		
Sulfadimethoxine	10	105		14.4	14.9	100		
	20	106	5.6	3.8	4.7	100	5	9
	30_(n=20)_	103		5	8.5	100		
Sulfadimidine	10	104		14.6	15	100		
	20	105	5.2	4.4	5	100	5	10
	30_(n=20)_	102		5.4	7.9	100		
Sulfadoxine	10	103		14.6	15.2	100		
	20	106	7.1	4.4	4.6	100	5	10
	30_(n=20)_	106		5.2	9.2	100		
Sulfamerazine	10	104		15.0	15.5	100		
	20	105	1.7	3.9	4.2	100	5	9
	30_(n=20)_	103		4.6	6.8	100		
Sulfamethizole	10	98		14.4	14.9	100		
	20	103	3.0	5.8	5.9	100	5	9
	30_(n=20)_	101		4.9	8.4	100		
Sulfamethoxazole	10	107		16.5	17.4	100		
	20	108	19.5	6.5	8.7	100	6	10
	30_(n=20)_	105		6.5	11.2	100		
Sulfamethoxypyridazine	10	99		15.8	16.3	100		
	20	105	3.1	5	5.1	100	4	9
	30_(n=20)_	104		6.1	8.9	100		
Sulfamoxole	10	101		21.1	22.4	100		
	20	105	2.7	14.4	16.9	100	<10	10
	30_(n=20)_	98		20.4	25.8	100		
Sulfaphenazole	10_(n=20)_	93		13.5	15.2	95		
	20	93	4.7	10.3	11.7	100	<10	10
	30_(n=20)_	95		13.6	18.2	100		
Sulfapyridine	10	102		14.2	14.2	100		
	20	104	5.1	4.1	5.3	100	5	10
	30_(n=20)_	99		5.7	8.4	100		
Sulfaquinoxaline	10	106		16.5	19.5	100		
	20	105	7.5	9.5	9.6	100	8	10
	30_(n=20)_	101		9.5	10	100		
Sulfathiazole	10	103		16.3	16.5	100		
	20	104	2.7	5.8	5.8	100	5	10
	30_(n=20)_	101		4.7	6.7	100		
Sulfisoxazole	10	103		15.3	15.8	100		
	20	103	5	5.2	5.5	100	6	10
	30_(n=20)_	101		5.9	10.1	100		
Sulfamonomethoxine	10_(n=20)_	112		10.8	11.2	100		
	20	112	7.6	8.3	9	100	<10	10
	30	116		25	26.5	100		

Compounds indicated in grey do fully comply with the quantitative criteria

^a^ CCα and CCβ for the compounds in grey were determined using the linearity approach; for the other compounds, CCβ was set to the first concentration level with 95% confirmed identity.

^−^ Underlined values do not comply with the criteria established in 2002/657/EC. Compounds in grey are applicable for quantitative confirmatory analysis

#### Qualitative performance

There were no interfering signals in the blank samples at the retention times corresponding to the product ions of any of the validated compounds. The selectivity of the method is therefore considered to be sufficient. After 28 d of storage at −80 °C, the samples were re-analyzed and results were re-evaluated based on the qualitative validation criteria. For the quantitative compounds, trueness was also re-evaluated. After 28 d, all compounds still complied with the qualitative and/or quantitative validation criteria and therefore it was concluded that the sample extracts are stable for at least 28 d if stored at −80 °C.

At all concentration levels, the identity for all compounds is confirmed in at least 95% of the samples, except for nalidixic acid and norfloxacin. For norfloxacin, the sensitivity of the second diagnostic ion was, for unknown reasons, low during the first validation day compared with the other days. This resulted in a higher error and for three out of the seven samples the identity could not be confirmed. For nalidixic acid the peak shape was suboptimal during the first day of validation. This resulted in an ion ratio deviation that is slightly higher than permitted according to the criteria. On validation d 2 and d 3 all additions in all samples satisfy the ion ratio criteria. Based on the results on d 2 and d 3, it is expected that all antibiotics can be detected and their identity can be confirmed using the applied method at the indicated concentration levels.

#### Quantitative performance

For tetracyclines, trueness lies between 85 and 106%, for quinolones between 93 and 131%, for macrolides between 73 and 134%, for lincosamides between 99 and 107%, for pleuromutilines between 103 and 129%, and for sulfonamides between 93 and 116%.

Linearity was determined using a calibration curve that was injected before and after the samples. For most compounds, linearity complies with the established criterion. For some compounds (tiamulin, sulfamethoxazole, tilmicosin, tylosin, ciprofloxacin, norfloxacin, and natamycin) a coefficient of correlation of <0.990 was found on one occasion; in most cases the calibration line at the end of a sample batch.

For all compounds, the RSD_r_ complies with the criteria, with the exception of sulfamethoxazole, pirlimycin, tylosin, and tylvalosin. This demonstrates that the method yields satisfactory quantitative performance for most compounds within a single batch of feathers. Note that for sulfamethoxazole, RSD_r*_ does comply with the criteria, indicating that the batch of feathers used for determination of RSD_r_ is a challenging one. This also demonstrates the importance of including a large number of different feather batches in a validation.

For 26 out of 45 compounds (58%), also the RSD_r*_ and RSD_RL_ comply with the criteria at the validation target level and higher. Most of these compounds also comply at 0.5*target level. Clearly, there is a large batch to batch variation, which is most likely the result of severe matrix effects, resulting in an increase of the analytical variation. Nevertheless, for these 26 compounds the method can be applied, not only for qualitative confirmatory analysis but also for quantification of the level of antibiotics present. Out of these 26 compounds, 25 compounds have an isotopically labeled standard available. Note that because repeatability of the method is sufficient, for the other 19 compounds the method can, besides only for qualitative confirmatory analysis, also be applied for quantitative analysis within a single batch as is appropriate for feather segmentation analysis [7, 12]. Quantitative performance could be further improved by using additional isotopically labeled internal standards, if commercially available.

CCα and CCβ were found to be equal or better than the lowest concentration level included in the validation. The CCα and CCβ for compounds that were found eligible for quantitative analysis were calculated using the linearity approach and are all below 0.5*target level. The CCα for the compounds that were not eligible for quantitative analysis are set to 0.5 * target level, the lowest validated concentration that still satisfied the confirmation criteria.

#### LOD and LOC for freely-extractable residues

In the analysis of freely extractable residues, approximately 1 g of feather sample is taken into account. This results in a significantly lower LOD and LOC compared with the analysis of ground feathers (sample intake 100 mg). Therefore, additionally the LOD and LOC were determined for this efficient, effective qualitative approach.

Most compounds show a LOD of 2.0 ng per portion of feathers (approximately 1 g) or lower. The LOD was higher for sulfachloropyridazine (2.5 ng), tylosin (10 ng), and tylvalosin (20 ng). Most compounds have a LOC of 2.0 ng or lower. The LOC was higher for sulfachloropyridazine (2.5 ng), neospiramycin (5 ng), oxytetracycline, erythromycin, tylosin, valnemulin (10 ng), and tulathromycin, tylvalosin (60 ng). Because data was unavailable, it was unknown if these detection limits were adequate for effective analysis of antibiotics on feathers. Therefore, this procedure was applied to real samples.

### Analysis of feather samples

A total of 20 feather samples of chickens for which antibiotic treatment was registered in the food chain information (FCI) were obtained from a slaughterhouse. In total, 26 different medicines (of which the active compounds are within the scope of this paper) were administered. The feather samples were analyzed for freely extractable residues and compared with the registered treatments according to the FCI. An overview of this comparison is presented in Table 4. In 23 out of the 26 treatments, the active compounds were successfully detected. Note that some of the treatments that were detected during slaughter occurred, according to registration, over a month before slaughter.

**Table 4:** An overview showing the comparison between the administrated treatments according to registration of the farmer in the food chain information and the results of the feather analysis of freely extractable residues

Chicken #	Treatment					Detected freely extractable residues	
	Medicine	Active compound(s)	Start of treatment (days before slaughter)	No. of days treated	Concentration (mg kg^−1^ body weight/d)	Component	Amount (ng g^−1^)
1	Spectron, Laboratorios Hipra S.A. (Zul.-Nr. 401356.00.00)	Enrofloxacin	42	3	10	Enrofloxacin	49
						Ciprofloxacin	5
2	Pyanosid Pulver, Bela-Pharm (Zul.-Nr. 13076.00.00)	Lincomycin/Spectinomycin	42	3	16.6 / 33.3	Lincomycin	4
3	Lanflox, Dopharma Research (Zul.-Nr. 401347.00.00)	Enrofloxacin	34	3	10	-	-
	Sulfadimidine Na, Eurovet Animal Health BV (Zul.-Nr. 6093467.00.00)	Sulfadimidine	27	6	100	Sulfadimidine	5300
	Pyanosid Pulver, Bela-Pharm (Zul.-Nr. 13076.00.00)	Lincomycin/Spectinomycin	30	3	16.6 / 33.3	Lincomycin	3
4	Lanflox, Dopharma Research (Zul.-Nr. 401347.00.00)	Enrofloxacin	34	3	10	-	-
	Sulfadimidine Na, Eurovet Animal Health BV (Zul.-Nr. 6093467)	Sulfadimidine	27	6	100	Sulfadimidine	4400
	Pyanosid Pulver, Bela-Pharm (Zul.-Nr. 13076.00.00)	Lincomycin/Spectinomycin	30	3	16.6 / 33.3	Lincomycin	4
5	Lincomycine 20%, Dopharma Research (REG NL 3095)	Lincomycin	28	3	20-30	Lincomycin	6
6	Tylo-Suscit, Bela-Pharm (Zul.-Nr. 6933163.00.00)	Tylosin	11	3	100	Tylosin	234*
7	Methoxasol-T, Dechra Veterinary Products (Zul.-Nr. 401190.00.00)	Sulfamethoxazole/Trimethoprim	45	4	33	Sulfamethoxazole	15
8	T.S.-Sol 20/100, Dopharma Research (REG NL 7611)	Sulfamethoxazole/Trimethoprim	21	3	37.5	Sulfamethoxazole	3
9	T.S.-Sol 20/100, Dopharma Research (REG NL 7611)	Sulfamethoxazole/Trimethoprim	21	3	37.5	Sulfamethoxazole	4
10	Tylo-Suscit, Bela-Pharm (Zul.-Nr. 6933163.00.00)	Tylosin	11	3	100	Tylosin	1176*
11	T.S.-Sol 20/100, Dopharma Research (REG NL 7611)	Sulfamethoxazole/Trimethoprim	38	3	37.5	Sulfamethoxazole	76
12	Spectron, Laboratorios Hipra S.A. (Zul.-Nr. 401356.00.00)	Enrofloxacin	34	3	10	Enrofloxacin	17
13	Cosumix plus, Elanco Europe (REG NL 5388)	Sulfachloropyridazine/Trimethoprim	8	3	30	Sulfachloropyridazine	1858
14	Methoxasol-T, Dechra Veterinary Products (Zul.-Nr. 401190.00.00)	Sulfamethoxazole/Trimethoprim	46	4	33	Sulfamethoxazole	7
						Sulfadimidine	4
15	Tylan W.O., Elanco Europe (REG NL 9984)	Tylosin	16	3	20-100	Tylosin	237*
16	Enro Sleecol, Eurovet Animal Health BV (Zul.-Nr. 401098)	Enrofloxacin	40	3	5	Enrofloxacin	29
						Ciprofloxacin	4
	Tylo-Suscit, Bela-Pharm (Zul.-Nr. 6933163.00.00)	Tylosin	30	2	100	Tylosin	-
17	Enro Sleecol, Eurovet Animal Health BV (Zul.-Nr, 401098)	Enrofloxacin	40	3	5	Enrofloxacin	11
	Tylo-Suscit, Bela-Pharm (Zul.-Nr. 6933163.00.00)	Tylosin	30	2	100	Tylosin	13*
						Lincomycin	3
18	T.S.-Sol 20/100, Dopharma Research (REG NL 7611)	Sulfamethoxazole/Trimethoprim	42	3	37.5	Sulfamethoxazole	17
19	Doxylin 50% WSP, Dopharma Research (REG NL 8753)	Doxycycline	10	3	25	Doxycycline	535*
20	Doxylin 50% WSP, Dopharma Research (REG NL 8753)	Doxycycline	10	3	25	Doxycycline	715*

*Estimated amount for qualitative compounds.

In three cases, antibiotics were administered but could not be detected. Two relate to a Lanflox treatment (active compound is enrofloxacin, samples 3 and 4) that was carried out 31 d before slaughter. These samples were obtained from the same farmer, but from different stables. Note that in sample 1, enrofloxacin was detected 39 d after the end of the treatment. For sample 1, Spectron was used and for samples 3 and 4, this is Lanflox. However, this does not explain the difference because both are applied at the same dosage and for 3 d. The other relates to the use of Tylo-Suscit (active compound is tylosin), 29 d before slaughter (sample 16). Note that another treatment with Tylo-Suscit (sample 17), also 29 d before slaughter, was detected with a concentration just above the detection limit of tylosin. Therefore, it could be possible that the concentration of tylosin in sample 16 was just below the detection limit and as a result not detected.

In two cases, ciprofloxacin was detected. These animals were treated with enrofloxacin and as a result its metabolite ciprofloxacin is present in low concentrations as well. Furthermore, in two cases, antibiotics were detected in the feathers even though, according to the registration, they were not administered. This regards sample 14, in which sulfadimidine was detected in addition to sulfamethoxazole, and sample 18, in which a low concentration of lincomycin was detected next to tylosin.

These samples demonstrate that the presented method successfully detects most antibiotics that are administered to poultry, even over a month after treatment. The method proves to be a strong tool in the enforcement of the correct registration of antibiotic administration in the poultry sector.

## Conclusion

A qualitative confirmatory method was validated for the analysis of tetracyclines, sulfonamides, quinolones, macrolides, lincosamides, and pleuromutilines in chicken feathers. The method is applicable for qualitative confirmatory analysis for all compounds included and, additionally, 58% of the compounds can also be analyzed quantitatively showing trueness, repeatability, and within-laboratory reproducibility within the criteria established in CD 2002/657/EC. Additionally to analyzing the total residue concentration in feathers, we propose a more cost-efficient method for analysis of freely extractable residues only. With this approach, antibiotic use in the poultry sector can effectively be monitored; in many cases even if the animals were treated in the first week of their lives and samples were taken at slaughter.
